# Metabolic and evolutionary patterns in the extremely acidophilic archaeon *Ferroplasma acidiphilum* Y^T^

**DOI:** 10.1038/s41598-017-03904-5

**Published:** 2017-06-16

**Authors:** Olga V. Golyshina, Hai Tran, Oleg N. Reva, Sofia Lemak, Alexander F. Yakunin, Alexander Goesmann, Taras Y. Nechitaylo, Violetta LaCono, Francesco Smedile, Alexei Slesarev, David Rojo, Coral Barbas, Manuel Ferrer, Michail M. Yakimov, Peter N. Golyshin

**Affiliations:** 1 School of Biological Sciences, Bangor University, LL57 2UW Bangor, Gwynedd, UK; 2 Centre for Bioinformatics and Computational Biology, Department of Biochemistry, University of Pretoria, Pretoria, 0002 South Africa; 3 Department of Chemical Engineering and Applied Chemistry, University of Toronto, M5S3E5 Toronto, Ontario Canada; 4 CeBiTec Bielefeld University, Universitätsstraße 25, D-33615 Bielefeld, Germany; 5 Insect Symbiosis Group, Max Planck Institute for Chemical Ecology, Hans-Knöll-Straße 8, D-07745 Jena, Germany; 6 Institute for Coastal Marine Environment, CNR, Spianata S. Raineri 86, 98122 Messina, Italy; 7 Fidelity Systems, Zylacta Corporation, 7965 Cessna Avenue, Gaithersburg, MD 20879 USA; 8 Centro de Metabolómica y Bioanálisis (CEMBIO), Facultad de Farmacia, Universidad CEU San Pablo, Campus Montepríncipe, Madrid Spain; 9 Institute of Catalysis CSIC, Campus Cantoblanco, 28049 Madrid, Spain; 10 Immanuel Kant Baltic Federal University, Universitetskaya 1, 36040 Kaliningrad, Russia; 11 Department of Bioinformatics and Systems Biology, Justus Liebig Universität Gießen, Heinrich-Buff-Ring 58, D-35392 Gießen, Germany

## Abstract

*Ferroplasmaceae* represent ubiquitous iron-oxidising extreme acidophiles with a number of unique physiological traits. In a genome-based study of *Ferroplasma acidiphilum* Y^T^, the only species of the genus *Ferroplasma* with a validly published name, we assessed its central metabolism and genome stability during a long-term cultivation experiment. Consistently with physiology, the genome analysis points to *F. acidiphilum* Y^T^ having an obligate peptidolytic oligotrophic lifestyle alongside with anaplerotic carbon assimilation. This narrow trophic specialisation abridges the sugar uptake, although all genes for glycolysis and gluconeogenesis, including bifunctional unidirectional fructose 1,6-bisphosphate aldolase/phosphatase, have been identified. Pyruvate and 2-oxoglutarate dehydrogenases are substituted by ‘ancient’ CoA-dependent pyruvate and alpha-ketoglutarate ferredoxin oxidoreductases. In the lab culture, after ~550 generations, the strain exhibited the mutation rate of ≥1.3 × 10^−8^ single nucleotide substitutions per site per generation, which is among the highest values recorded for unicellular organisms. All but one base substitutions were G:C to A:T, their distribution between coding and non-coding regions and synonymous-to-non-synonymous mutation ratios suggest the neutral drift being a prevalent mode in genome evolution in the lab culture. Mutations in nature seem to occur with lower frequencies, as suggested by a remarkable genomic conservation in *F. acidiphilum* Y^T^ variants from geographically distant populations.

## Introduction

*Ferroplasma acidiphilum* Y^T^ (DSM 12658^T^) from the family *Ferroplasmaceae*, order *Thermoplasmatales*, phylum *Euryarchaeota* are iron-oxidising extreme acidophiles that require small amounts (0.02% w/vol) of yeast extract for growth and populate environments with low pH values and rich in sulfur compounds and metals in the form of sulfides^1, 2^. Various ecological aspects related to this widely distributed archaeal group were reviewed earlier^3^. Deep metagenomic and metaproteomic investigations of microbial communities of acid mine drainage (AMD) biofilms in Iron Mountain (CA, USA) inhabited *inter alia* by the members of the family *Ferroplasmaceae*, have been conducted to provide some insights into, and hypotheses on, their metabolism and physiology^4–6^. A number of uncommon biochemical features have also earlier been revealed for *F. acidiphilum* Y^T^, such as an unusually high proportion of iron-containing proteins in the proteome and low pH optima for the enzyme activities *in vitro* ^7–9^. Despite aforementioned research milestones on *Ferroplasmaceae*, there is a further need in investigation of metabolism of *F. acidiphilum* Y^T^, important in the relation to the practical applications and for filling the void in our understanding of fundamental mechanisms of its lifestyle. In particular, there is still no consensus on the major mechanisms of carbon assimilation and hence on the major role of *Ferroplasma* spp. play in the environment (apart from the ferrous oxidation, which is well established and characterised in detail). Suggested patchiness of the genomic pools of, and frequent recombinations in genomic variants in *Ferroplasma* spp. and “*Ferroplasma acidarmanus*” fer1 in their natural environment^10^ that could also be linked with a certain mosaicness of assemblies resulting from metagenomic data from a multitude of clonal variants, could also be verified by the analysis of a genome from geographically distant, yet closely related sibling with 100% SSU rRNA gene sequence identity. For this, the high-quality, ungapped genome from a characterised reference isolate from a similar environment represents a good opportunity.

Here, we present the genome-based and wet-lab analysis of *F. acidiphilum* Y^T^ in the context of its niche adaptation, nutrients acquisition, energy and carbon metabolic pathways and its relatedness with phylogenetic neighbours. Furthermore, we provide an overview of the *in vitro* genome evolution patterns during the long-term maintenance of the strain in the laboratory culture.

## Results and Discussion

### Genome stability and evolution

#### General genome features

The size of the genome of *F. acidiphilum* Y^T^ is 1.826.943 bp, G + C content 36.49%, the total gene number was predicted to be 1773 (excluding 19 CDS with pseudogene qualifiers) with a coding density of 86.4%; 508 genes were revealed to code for hypothetical proteins. Loci for 5S, 16S and 23S rRNA are not arranged in a single operon, but scattered in the chromosome; 46 tRNAs were predicted.

#### Genome sequence comparison of *F. acidiphilum* Y^T^ with “*F. acidarmanus*” strain fer1

Strains *F. acidiphilum* Y^T^ and *“F. acidarmanus”* fer1 have zero mismatches in their 16S rRNA gene sequences, which, nevertheless, does not prove by itself that both belong to the same species. It was therefore worth to assess their relatedness by using the Average Nucleotide Identity (ANI) analysis (http://enve-omics.ce.gatech.edu/ani/ ^11^). The analysis suggested the median ANI value of 98.7% (Fig. S1), which is well above the commonly accepted cut-off (95%) for separation of two species based on the whole-genome comparisons. In addition to that, the application of the online Genome-to-Genome Distance Calculator (GGDC 2.0 tool, http://ggdc.dsmz.de/distcalc2.php ^12^) using all three default calculation formulae suggested DNA-DNA hybridization (DDH) values 73.1, 85.5 and 85.80% and DDH values >= 70% with the probabilities 83.97, 97.3 and 98.38, correspondingly. To sum up, both analyses suggested that based on their genomic sequences, *F. acidiphilum* Y^T^ and “*F. acidarmanus*” belong to the same species, despite showing some physiological differences reported earlier^5^. Interestingly, the geographical separation of these two organisms (and many others, as one can judge from metagenomic assemblies in public sequence databases) has not lead to a great deal of speciation. This may also suggest that their geographical separation occurred relatively recently and that despite the affiliation of these archaea to a very special niche, they must be rather robust to, and persistent in the, non-acidic environments, which allows them to disseminate and colonise the sulfidic, low-pH niches across the planet. Seemingly under natural conditions the evolution of such small genome-sized (and hence having a narrow metabolic repertoire), slowly metabolising organisms is on-going at lower rates, which restricts the genome evolution and therefore prevents the divergence and speciation. This is also in line with the suggestion that small and compact genomes, as well as single-copy rRNA genes are the signs for minimising metabolic costs in habitats where neither a broad metabolic repertoire, nor high numbers of paralogous proteins are needed to accommodate growth under very constant and stagnant environmental conditions.

#### Horizontally transferred genomic islands in *F. acidiphilum* Y^T^

**Figure 1:**
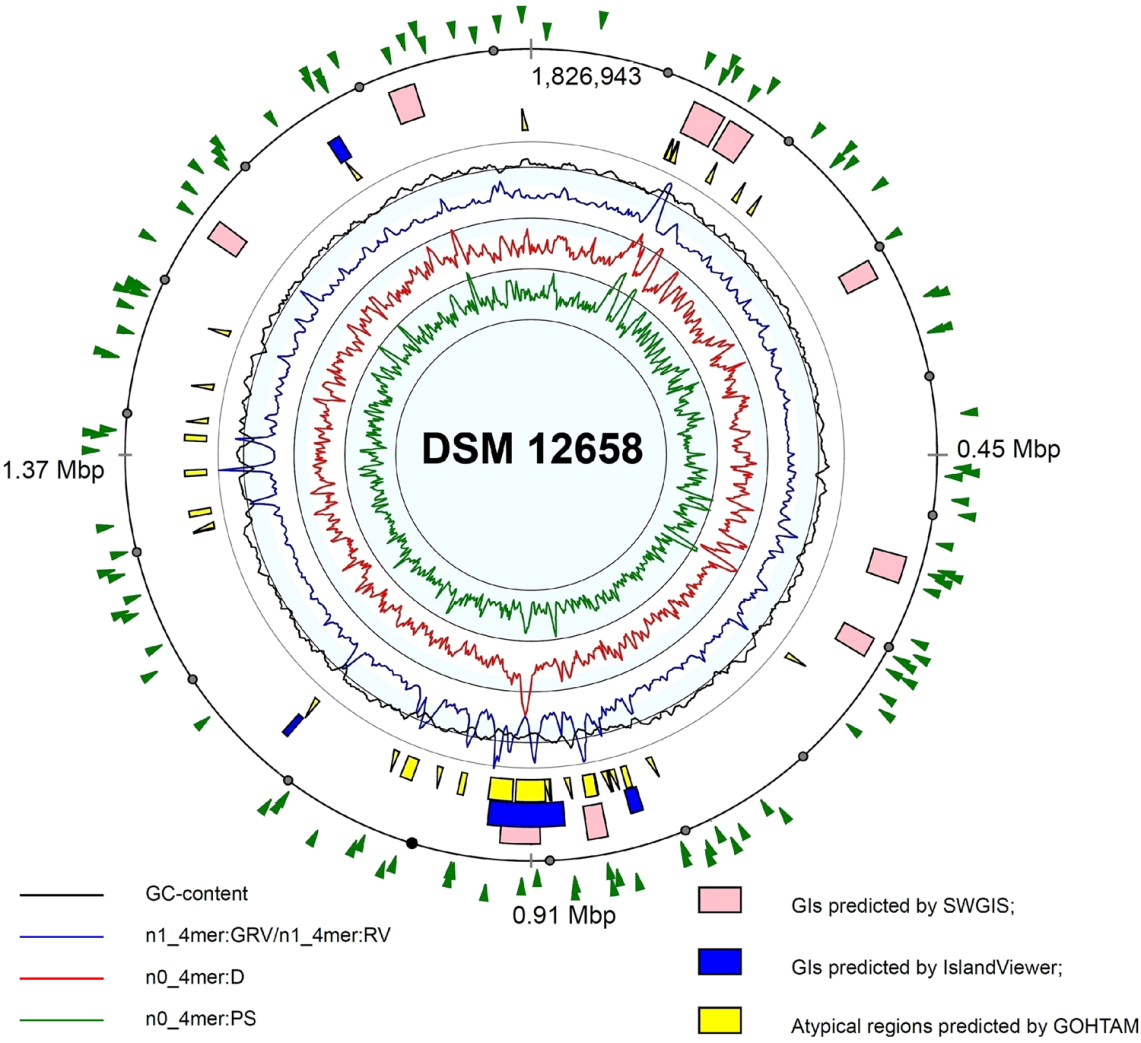
The genome and genomic islands (GI) of *F. acidiphilum* Y^T^. Localization of GIs on the chromosome of *F. acidiphilum* Y^T^, as predicted by SWGIS (pink boxes), IslandViewer (blue boxes) and GOTHAM (yellow boxes). Histograms in the inner cycles of the atlas depict variations of the following oligonucleotide usage parameters: GC-content (black curve); ratio of generalized to local relative variances calculated for tetranucleotide usage patterns normalized by the GC-content (blue curve, n1_4mer:GRV/n1_4mer:RV); distances between not-normalized local tetranucleotide usage pattern and the global one calculated for the complete chromosome (red curve, n0_4mer:D); asymmetry between not-normalized tetranucleotide usage patterns calculated for the direct and complement DNA strands (green curve, n0_4mer:PS). Use of these parameters for GI identification and their standard abbreviations were explained in more detail^68^. Green arrowheads (outer circle) indicate single-nucleotide substitutions in the genome of *F. acidiphilum* Y^T^ after ~550 generations in the laboratory culture (s. Supplementary Table S2 for more details).

Horizontally transferred genomic islands (GIs) were identified in the complete genome sequence of *F. acidiphilum* Y^T^ by the Seqword Gene Island Sniffer (SWGIS) program^13^, IslandViewer program package comprising three different GI prediction algorithms^14^ and by GOHTAM^15^. Joint results of GI identification by different methods are shown in Fig. 1. Nine putative GIs characterized by alternative oligonucleotide usage (OU) patterns were detected by SWGIS and IslandViewer programs predicted three shorter GIs. GOHTAM returned many short regions with atypical tetranucleotide and/or codon usage; however, not all of them necessarily were of a lateral origin. Predicted GIs mostly harboured genes with unknown functions, a few transposases and several enzyme-coding genes including a gene cluster of archaeal sulfocyanin-containing respiratory system and a beta-lactamase in GI [126,000–156,681] and a cluster of genes encoding CRISPR-associated proteins in seventh GI [905,732–938,099] (see below for more details). Our findings indicate that the horizontal gene transfer might play an important role in the evolution of metabolic pathways of *F. acidiphilum* Y^T^ and in the acquisition of a resistance against viruses.

GIs identified were searched for tetranucleotide pattern similarity through the database of 17,984 GIs detected in 1,639 bacterial and chromosomal sequences (see the database at www.bi.up.ac.za/SeqWord/sniffer/gidb/index.php)^16^. Significant compositional similarity of GIs from *F. acidiphilum* Y^T^ was found with GIs of many other archaea and bacteria belonging to distant taxonomic units. However, the highest similarity was observed between GIs of *F. acidiphilum* and another acidophilic archaeon *Thermoplasma volcanium* GSS1. Remarkably, among recipients of GIs from other extremophiles, there were several *Bacteroides* species.

The factor playing an important role in the genome evolution and lateral gene transfer are transposases. In total, 80 transposases have been predicted, among them 28 belonged to IS4 family proteins and 10 were affiliated with MutS transposase mutator family proteins (COG3328). As it was suggested earlier^17^ the MutS homologs are abundant in *Euryarchaeota* and could be indicative to the gene transfer from bacteria to archaea. Other genes encode IS605 OrfB family transposases and ISA0963 transposases, IS2000 family protein, MULE, OrfA of protein families, consistently with the previous reports of *Thermoplasmatales* to commonly carry numerous ISs of the families IS4 IS5, IS256, IS481, ISA1214 and IS2000/605/607^18^.

#### Mismatch repair and recombination

Recombination and mismatch repair proteins were represented by the DNA resolvase (FAD_0665) exhibiting a relatively low similarity to its counterparts from methanogens and bacteria. DNA-repair helicase FAD_1466 was similar to archaeal Rad25 proteins, FAD_1503 exhibited 30% identity with *Sulfolobales* XPD/Rad3-related DNA helicases and with another DNA repair protein FAD_1564. Genes FAD_0550 and FAD_0559 encode DNA repair and recombination proteins RadA and RadB, archaeal homologs of RecA and Rad51, respectively; the latter is considered of being *Euryarchaeota-*specific^19^. Mismatch repair proteins, MutS-like ATPases (FAD_0765–0766), were most similar to MutS proteins from bacteria and *Thermoplasmatales*. The genome encodes a number of endonucleases, namely of the type II restriction endonuclease FAD_0313 exhibiting a high similarity only with bacterial proteins, two gene copies for endonucleases of types III (FAD_1157, 1370), IV (FAD_0129, 1301) and of type V (FAD_0403) as well as Fen1 (FAD_0558) and PolX (FAD_1333) endonucleases.

#### Clustered Regularly Interspaced Short Palindromic Repeats (CRISPR)

**Figure 2:**
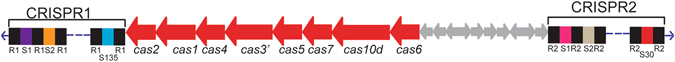
Clustered Regularly Interspaced Short Palindromic Repeats (CRISPR) locus in *F. acidiphilum* Y^T^ with one operon encoding the CRISPR-associated (Cas) proteins (red arrows). CRISPR system belongs to the Subtype I-D. Ten genes not related to CRISPR are shown in grey. The cluster CRISPR1 contains 137 identical direct repeats of 30 bp separated by 136 different spacers of 34–39 bases. The Cluster CRISPR2 is shorter and has 31 direct repeats (31 bp each) with 30 different spacers (35–62 nt). Spacers and repeats in Clusters 1 and 2 show no sequence similarity one to another.

The *F. acidiphilum* Y^T^ genome revealed the presence of two clusters of Clustered Regularly Interspaced Short Palindromic Repeats (CRISPR) separated by one operon encoding the CRISPR-associated (Cas) proteins and ten genes, which are not related to CRISPR (Fig. 2). CRISPRs and Cas proteins represent a microbial small RNA-based interference system found in most archaea and many bacteria; the CRISPR-Cas system functions as the adaptive microbial immune system against invading viruses and plasmids, and it also has a role in microbial pathogenesis, DNA repair, and biofilms^20^. The cluster CRISPR1 of *F. acidiphilum* Y^T^ is quite large and contains 133 identical and 3 degenerated direct repeats (30 bp long) separated by 135 different spacers of similar size (34–39 bp) (Fig. 2). The cluster CRISPR2 is smaller with 31 direct repeats (31 bp each) separated by 30 different spacers (35–38 bp, with spacer 5 being 62 bp). Neither spacers, nor repeats from these clusters share any sequence similarity to each other. The rather large size of both CRISPR1 and CRISPR2 arrays might be indicative of high activity of the *F. acidiphilum* Y^T^ CRISPR system^21, 22^. The NCBI Blast analysis of the *F. acidiphilum* Y^T^ CRISPR spacers revealed no homologous sequences present in the available viral genomes or plasmids suggesting that its CRISPR targets have yet to be discovered. Only the spacer 2 from cluster CRISPR2 was found to be identical to a region in a gene encoding the hypothetical protein FACI_IFERC00001G0010 in the “*F. acidarmanus*” genome, a large uncharacterized protein with the predicted UvrD-like helicase and restriction endonuclease type II-like domains. Although the “*F. acidarmanus”* genome also encodes two CRISPR clusters and eight *cas* genes, their repeat sequences showed no similarity one to another suggesting that their CRISPR systems are not related. The eight *cas* genes of *F. acidiphilum* Y^T^ are associated with the cluster CRISPR1 and are expected to be co-transcribed (*cas6*, *cas10*, *cas7*, *cas5*, *cas3*, *cas4*, *cas1*, and *cas2*) (Fig. 2). Based on the *cas* gene arrangement and the presence of *cas10*, the *F. acidiphilum* Y^T^ CRISPR-Cas system can be classified as a CRISPR subtype I-D, which is similar to the type III system^23^. This is consistent with the fact that most archaea contain the CRISPR subtypes A, B, or D^24^. In most of the type I and III CRISPR systems, Cas6 proteins cleave long pre-CRISPR RNA (crRNA) transcripts generating mature crRNAs containing a single spacer with flanking repeat fragments^25^. Based on sequence, the type I-D CRISPR repeats have been predicted to form hairpin structures, which are recognized by Cas6 proteins and cleaved at the 3′-base of the stem-loop. Analysis of the *F. acidiphilum* Y^T^ CRISPR1 and CRISPR2 repeats revealed that they can form similar hairpin structures suggesting that both CRISPR1 and CRISPR2 pre-crRNAs can be processed by the single *F. acidiphilum* Y^T^ Cas6 protein. Comparison of amino acid sequences of the *F. acidiphilum* Y^T^ Cas proteins with GenBank identified the Cas1 and Cas2 proteins from *Picrophilus torridus* (an acidophilic archaeon and the closest phylogenetic neighbour of *Ferroplasmaceae*) as the top BLAST hits (50% and 46% sequence identity, respectively). However, other Cas proteins from *F. acidiphilum* Y^T^ were more similar to the corresponding Cas proteins from the metagenomic assembly dubbed “*Ferroplasma* sp. Type II” (58% to 75% sequence identity).

#### Analysis of mutations over the long-term cultivation *in vitro*

Comparison of two variants of genomes of *F. acidiphilum* Y^T^ (i.e. the original culture deposited in the DSMZ in 1998 (DSM 12658^T^) and the culture continuously grown in laboratory with re-inoculation intervals of 24.5 days for 11 years) revealed 116 single-nucleotide substitutions (see Supplementary Table S2 for details on substitutions and single-nucleotide polymorphism) randomly scattered across the chromosome (Fig. 1), green arrowheads on the outer circle). 115 out of 116 were GC to AT substitutions; such nucleotide shift is a common tendency for spontaneous single-base substitutional mutations^26^ and indicates that *F. acidiphilum* Y^T^ genome with already low GC content is prone to further AT enrichment. Among substitutions, 12 (about 11%) were detected in non-coding sequences that is consistent with the overall coding percentage (86.4%) in the genome. From bases’ substitutions in coding sequences, 34 of 103 (i.e. 33%) were synonymous. Majority of 69 non-synonymous base substitutions resulted in non-conservative amino acid changes and only in 7 cases resulted in conserved ones. 11 genes had two substitution sites (Table S2). Substitutions in coding regions mostly occurred in genes with known functions but also in 17 genes encoding hypothetical proteins (almost all these proteins contain one or more conserved domains). Some base substitutions occurred in genomic islands 1, 2 and 4, specifically in the GI 1, which contains gene clusters coding for ribosomal proteins (Fig. 1 and Table S2). Distribution of substitutions in other GIs showed evidence of those in functional genes, only one mutation occurred in a hypothetical gene.

**Figure 3:**
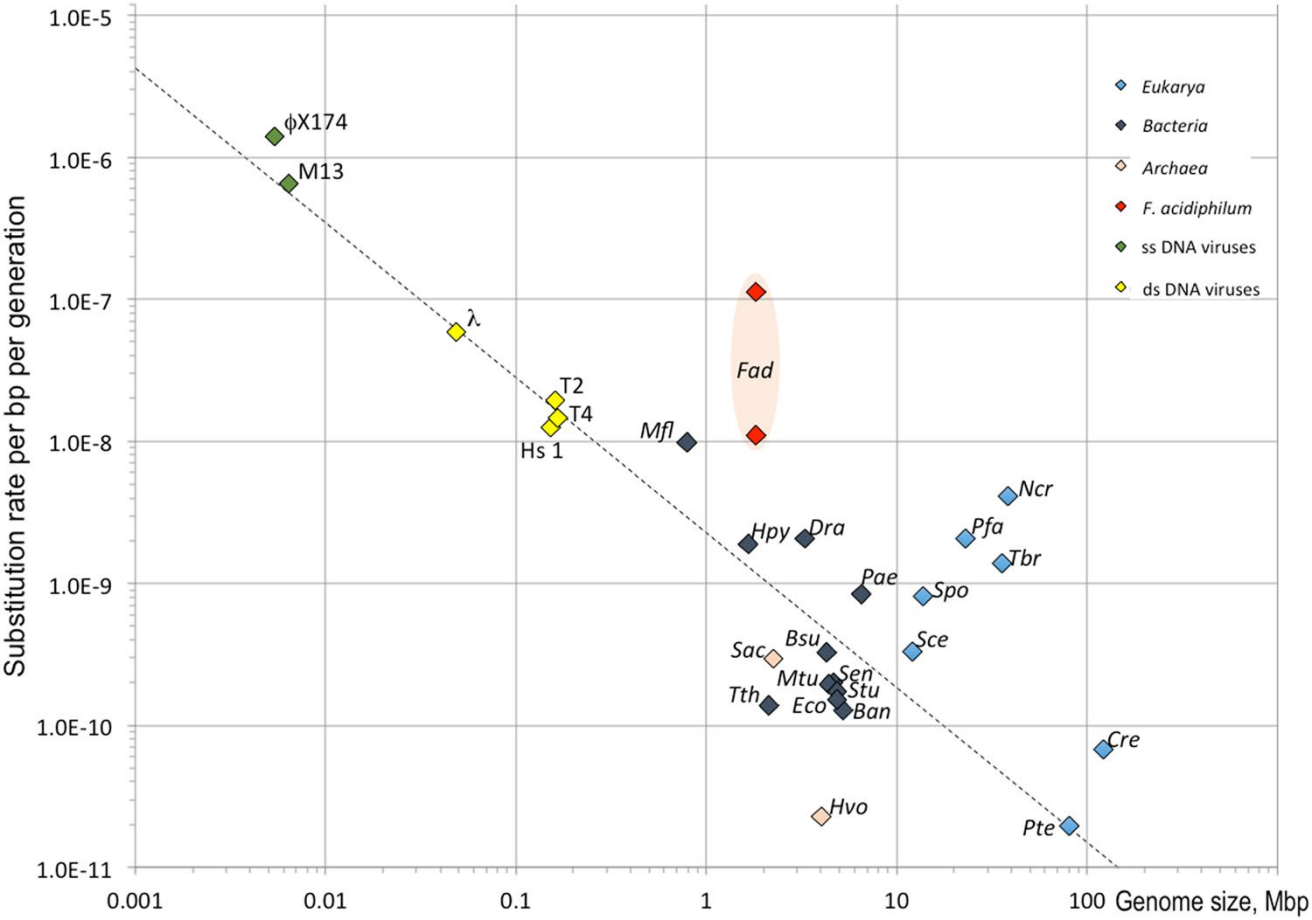
Single-nucleotide mutations accumulated during cultivation of *F*. *acidiphilum* Y^T^. Base-substitutional mutation rates per site per generation plotted *vs* genome sizes. The data on mutation rates of unicellular organisms and viruses and the regression plot (log_10_ *u* = *−*8.66–1.096log_10_G, where *u* and G are mutation numbers and genome size, respectively) are taken from^29^. Single-stranded DNA viruses: *ϕ*X174, phage *phi*174; M13, phage M13. Double-stranded DNA viruses: *ϕ*, phage lambda; T2, phage T2; T4, bacteriophage T4; Hs 1, Herpes simplex virus. Bacteria: *Bsu*, *Bacillus subtilis; Ban*, *Bacillus anthracis; Dra*, *Deinococcus radiodurans; Hpy*, *Helicobacter pylori; Mfl*, *Mesoplasma florum*; *Mtu*, *Mycobacterium tuberculosis; Pae*, *Pseudomonas aeruginosa; Sen*, *Salmonella enterica; Stu*, *Salmonella typhimurium; Tth*, *Thermus thermophilus*. Archaea: *Fad*, *Ferroplasma acidiphilum; Hvo*, *Haloferax volcanii; Sac*, *Sulfolobus acidocaldarius*. Eukarya: *Cre*, *Chlamydomonas reinhardtii; Ncr*, *Neurospora crassa; Pfa*, *Plasmodium falciparum; Sce*, *Saccharomyces cerevisiae; Spo*, *Schizosaccharomyces pombe; Tbr*, *Trypanosoma brucei; Pte*, *Paramecium tetraurelia*. Pink area reflects the distribution of mutation rates in *Ferroplasma* with the higher point value corresponding to all detected base substitutions in the strain cultured for ~550 generations as compared with the original genome, and lower value representing the most conservative mutation rate prediction (all mutations with frequency values <5% and SNPs in the original genome were excluded).

Base-substitutional mutation rate per nucleotide position per generation calculated for *F. acidiphilum* Y^T^ was within the highest range of that in other unicellular organisms, i.e. was similar or higher than that in *Mesoplasma florum* ^27^, which until now had the highest record of mutation rates per base per generation. According to the data^27^, in prokaryotic organisms, viruses and most (except four) unicellular eukaryotes base substitution rates per site per cell division fit the regression plot log_10_ *u = −*8.663–1.096log_10_G (*u* and G are mutation numbers and genome sizes, respectively, and *r* ^2^ = 0.872) (Fig. 3). *F. acidiphilum* Y^T^, however, occupies an outstanding position in this respect with remarkable 0.02 (conservative estimates) mutations per generation per genome (as a comparison, this figure for *Escherichia coli* is of approx. 0.001). We hypothesize that one of the possible reasons of these outstanding mutation rates may be the earlier observed abnormal abundance of intracellular iron in the cells of *F. acidiphilum* Y^T^ ^8^, which may under oxidative stress conditions be linked with excessive DNA damage by Fenton reaction. Another factor, which may contribute to the high mutation rates, is the error-prone DNA polymerase IV (FAD_1298), which is capable of inducing mutations at sevenfold higher rate than under its deficiency^28^. The experimental validation of above hypotheses though is yet to be conducted.

### Energy and carbon metabolism

#### Oxygen respiration and iron oxidation

The detailed biochemical study of the respiratory chain of *F. acidiphilum* strain Y^T^ has recently been reported^29^. Interestingly, the genes coding for electron flow chain involved in iron oxidation in *F. acidiphilum* Y^T^ were located in the identified Genomic Island/GI 1 (126,000–156,681), similarly to that in *P. torridus* and *Cuniculiplasma divulgatum*, where the origin of the respiratory complexes has also been attributed to the lateral gene transfer^30, 31^.

Related to the synthesis of the Fe-S systems we have detected the cysteine desulfurase gene (FAD_0633), co-clustered genes *sufC* and *sufB* genes (probably related to the above) and the hypothetical protein with a low similarity level to bacterial SufD like protein (FAD_1089–1087). There were 6 ORFs in the genome related to ferredoxin synthesis (FAD_0146 [COG0348]; FAD_0257 [COG1146]; FAD_1078 [COG1145]; FAD_1661 [COG2440]; FAD_1852 [COG1146] and FAD_1160 [COG2440]). Most of them contain 4Fe-4S clusters, providing low potential electron donors for redox processes in *F. acidiphilum* Y^T^.

#### Amino acids metabolism

Genome inspection of *F. acidiphilum* Y^T^ revealed incomplete synthesis pathways for histidine, isoleucine, leucine and valine (Fig. S2) pointing at the dependence on external sources and hence supporting the role of *Ferroplasma* in the environment as iron-oxidising proteolytic ‘scavengers’. The well-developed capacity for degrading amino acids is encoded by the *F. acidiphilum* Y^T^ genome. For example, we found the genes for the degradation of histidine via urocanate (FAD_1379) and tryptophane via kynurenine to anthranilate (FAD_0101–0104) and 2-oxoacid dehydrogenase complex (FAD_1290–1291). Transamination of aspartate and glutamate via aspartate aminotransferases (FAD_0393, 0538 and 1098) and glutamate dehydrogenase (FAD_0434) generates corresponding branched-chain 2-oxoacids, oxaloacetate and 2-oxoglutarate, which are citric acid cycle intermediates.

Bioleaching pilot plant, from where *F. acidiphilum* Y^T^ was isolated, contained ore particles of various sizes, where this archaeon may encounter anoxic microenvironments. Physiological studies performed on *F. acidiphilum* Y^T^ denoted this strain as a facultative anaerobe, coupling chemoorganotrophic growth on yeast extract to the reduction of ferric iron^5^. However, the detected reduction cannot be recognised as respiratory reactions since the obtained biomass was very low and close to no substrate-control. Nevertheless, we looked for corresponding genes relevant to a certain metabolic activity of *F. acidiphilum* Y^T^ strain under anaerobic conditions. Pyruvate can be converted to acetyl-CoA by a ferredoxin-dependent pyruvate oxidoreductase (POR, FAD_0567–0568). Obtained product may be converted to acetate by an ADP-forming acetyl-CoA synthetase thus providing substrate level phosphorylation step of pyruvate fermentation. Additionally, the *F. acidiphilum* Y^T^ genome possesses all genes necessary for complete arginine fermentation, i.e. arginine deiminase pathway. This ‘ancient’ catabolic route, converting arginine to ornithine, carbon dioxide, ATP and ammonium constitutes a major source of energy for some obligate anaerobic bacteria and fermenting archaea^32, 33^. Produced ammonium increases the intracellular pH and has been shown to be important for survival of various prokaryotes in acidic environment^34^. The arginine deiminase pathway was probably present in the last universal common ancestor (LUCA) to all the domains of life and its genes evolved independently, undergoing complex evolutionary changes leading to a later assemblage into a single cluster with functional interdependence^33^. It must be noted that all three genes of the arginine deiminase pathway, namely arginine deiminase (FAD_0428), ornithine transcarbamoylase (FAD_1523) and carbamate kinase (FAD_0067) are not in a single operon, but are located distantly one from another in the *F. acidiphilum* Y^T^ genome; the above has so far not been detected in any other but very closely related extremely acidophilic archaea.

Arginine fermentation route is not the only signature of ancient anaerobic LUCA metabolism, which occurs in the *F. acidiphilum* Y^T^ genome. Following the method described elsewhere^35, 36^, we identified several other genes of the ancient metabolic core including 6 methyltransferases (FAD_0113, 0367, 1012, 1218, 1562 and 1651), 5 SAM-dependent methyltransferases (FAD_0758, 0931, 1052, 1315 and 1729) and ferredoxin (FAD_0146) in addition to several subunits of the H^+^/Na^+^-antiporter Mrp/hydrogenases and related complexes (FAD_0579–0584). The acquisition of this antiporter comparable to [NiFe] hydrogenases was proposed as a crucial step at the early stages of bio-energetic evolution, which allowed conversion of geochemical pH gradient into the biologically more useful Na^+^ gradient^37^. Noteworthy, all these protein families are typical for strict anaerobes and rarely occur in genomes of aerotolerant or facultatively anaerobic prokaryotes, harbouring heme-copper oxygen reductases^36^. *F. acidiphilium* Y^T^ can be an example of such rare organisms that possess both LUCA candidate gene protein families alongside the cytochrome oxidases.

#### TCA cycle in *F. acidiphilum* Y^T^

**Figure 4:**
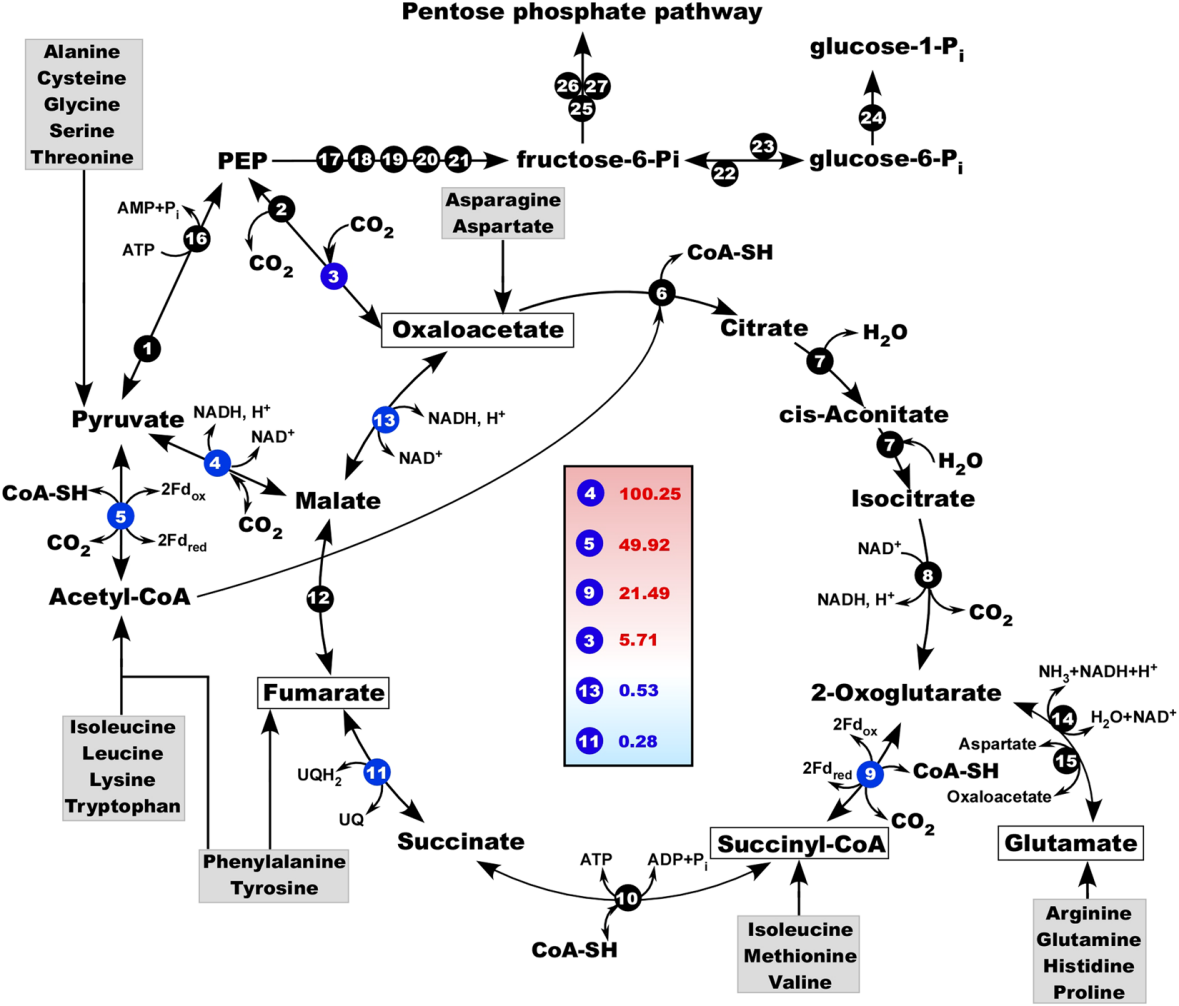
Proposed citric acid cycle and related enzyme reactions in *F. acidiphilum* Y^T^. The enzymes are as follows: 1, pyruvate kinase (FAD_1603); 2, PEP carboxykinase (FAD_1050); 3, PEP carboxylase (FAD_1044); 4, NAD-binding malic enzyme/malate dehydrogenase (FAD_0703); 5, pyruvate: ferredoxin oxidoreductase (FAD_0567–0568); 6, citrate synthase (FAD_1100); 7, aconitate hydratase (FAD_0701); 8, isocitrate dehydrogenase (FAD_1632); 9,2-oxoglutarate:ferredoxin oxidoreductase (FAD_0712–0713); 10, succinyl-CoA synthetase (FAD_0709–710); 11, succinate dehydrogenase (FAD_0714–0717); 12, fumarate hydratase (FAD_1630); 13, malate dehydrogenase (FAD_0718); 14, glutamate dehydrogenase (FAD_0434); 15, aspartate aminotransferase (FAD_1098); 16, phosphoenolpyruvate synthase (FAD_1233); 17, phosphoglycerate mutase (FAD_0440, FAD_1169, FAD_1350); 18,2-phosphoglycerate kinase (FAD_1810); 19, glyceraldehyde-3-phosphate dehydrogenase (FAD_0549); 20, triosephosphate isomerase (FAD_0107); 21, fructose-2,6-bisphosphatase (FAD_0332); 22,6-phosphofructokinase (FAD_0353); 23, bifunctional phosphoglucose/phosphomannose isomerase (FAD_0562); 24, phosphoglucomutase/phosphomannomutase (FAD_0602); 25, transketolase (FAD_1477–1476); 26, transaldolase (FAD_1201; FAD_1475); 27, ribulose-phosphate 3-epimerase (FAD_0295). Abbreviations used: Fd, electron carrier ferredoxin; NAD, nicotinamide adenine dinucleotide; CoA, Coenzyme-A; PEP, phosphoenolpyruvate; UQ, ubiquinone. Enzymes labeled in blue are potentially involved in anaplerotic assimilation of CO_2_. Their relative expression levels, analysed by RT-qPCR and indicated by the numbers in the central box, were obtained by normalization of the total RNA added and using transcripts of DNA gyrase subunit B (*gyr*B) as the internal reference (value 1.0). Normalization using *gyr*B was additionally validated *vs* transcripts of gene for ribosomal L2 protein. Average normalisation data derived from triplicates with standard deviation below 5%.

As observed in a multitude of studies, for a successful isolation of many prokaryotes, and especially archaea, the yeast extract should be added into the cultivation media as an essential component and a source of numerous cofactors and nutrients but also oligopeptides and amino acids. These nutrients are fundamental substrates feeding many metabolic pathways, including tricarboxylic (citric) acid cycle (TCA). This cycle is likely the central metabolic hub of *F. acidiphilium* Y^T^, while most proteins involved in the canonical TCA cycle were identified in genome, except for 2-oxoglutarate (OG) dehydrogenase complex (Fig. 4). In common with some other archaea^38, 39^, the conversions of pyruvate to acetyl-CoA and of 2-OG to succinyl-CoA are catalysed by the respective pyruvate:ferredoxin oxidoreductase (POR, FAD_0567–0568) and alpha-ketoglutarate:ferredoxin oxidoreductase (KOR, FAD_0712–0713). Although both enzymes were initially characterised as extremely oxygen-sensitive, POR and KOR activities have been demonstrated also in a number of obligately aerobic organisms^40, 41^. Compared to their anaerobic counterparts, these enzymes are oxygen-tolerant, exhibit lower rates and have an unusual subunit structure^42, 43^. Noteworthy, it has been suggested^44^ that to support biosynthetic reactions some aerobic prokaryotes might utilise KOR for the reductive carboxylation of succinyl-CoA to 2-OG. Given that succinyl-CoA synthetase, succinate dehydrogenase, fumarate hydratase and malate dehydrogenase are the enzymes that catalyse reversible reactions, the formation of 2-OG from oxaloacetate via malate, fumarate, succinate and succinyl-CoA is apparently plausible for *F. acidiphilum* Y^T^ (Fig. 4). This finding suggests that, while relying primarily on amino acids catabolism for carbon, *F. acidiphilium* Y^T^ can recruit the partially reverse, or reductive, TCA cycle as the additional anabolic strategy to produce important precursors for biosynthesis. This strategy was demonstrated in a number of archaea and acidophilic bacteria^45^.

Although we did not quantify the expression of all genes involved in TCA cycle, the transcriptomic analysis of succinate dehydrogenase and malate dehydrogenase revealed that both enzymes were expressed to a similar extent (Fig. 4). Recently, these enzymes were identified in *Ferroplasma* proteome as proteins induced during anaerobic growth coupled with ferric iron reduction^46, 47^. It is therefore most likely that under these conditions, in order to provide the terminal electron acceptor (Fe^3+^) with reducing power, the catabolic function of TCA cycle prevails over the anabolic.

In connection with the inability to use acetate as the sole carbon source, the key enzymes of the glyoxylate cycle, isocitrate lyase, and malate synthase, could not be identified in the *F*. acidiphilum Y^T^ genome. Concluding the description for the oxidative, partially “anaerobic” TCA cycle, it becomes apparent that due to the capability of KOR for the reductive carboxylation, *F. acidiphilum* Y^T^ cells possess an enzymatic machinery permitting to convert succinyl-CoA into 2-OG while fixing inorganic carbon. 2-OG can be directly converted into amino acids by glutamate dehydrogenase (FAD_0434), which assimilates ammonium and besides biosynthetic function can be regarded as a part of nitrogen metabolism. Additionally, glutamate can be also formed from 2-OG by an aspartate aminotransferase (FAD_1098) yielding oxaloacetate (Fig. 4).

#### Glycolysis/Gluconeogenesis

Growth on amino acids requires a gluconeogenic pathway for carbohydrate synthesis^48^ and in line with that all genes for a reverse glycolytic pathway have been identified. Interestingly, *Ferroplasma* possesses a gene encoding a bifunctional gluconeogenetic fructose 1,6-bisphosphate aldolase/phosphatase, a strictly anabolic enzyme, which is discussed as being an ancestral enzyme type^49^. Consistently, homologues for classical (glycolytic) fructose 1,6-bisphosphate aldolases are missing. Although *F. acidiphilum* Y^T^ was reported to be unable to use sugars as the sole carbon source, genes coding for some essentially irreversible reactions of glycolysis, besides aldolase, appear to be present in the genome. These are glucokinase (FAD_0277), phosphofructokinase (FAD_0353). Thus, it is likely that the absence of corresponding transporters preclude the uptake of external glucose, which, nevertheless, can be metabolised in phosphosugars and pentoses if synthesised *de novo* by *F. acidiphilum* Y^T^ cells. In consistency with findings in other archaea^50^, the oxidative pentose phosphate pathway is lacking in *F. acidiphilum* Y^T^, but its reductive part is fully present and likely operative (Fig. 4).

#### Putative CO_2_ assimilation mechanisms through gene expression analysis

Earlier it was reported that *F. acidiphilum* Y^T^ was able to incorporate into its biomass the inorganic carbon in the form of ^14^CO_2_^1, 51^. The genome analysis however did not suggest a clear assimilatory pathway whereas a number of carboxylation reactions may have led to the incorporation of CO_2_ into the biomass. Besides mentioned above reductive carboxylation of succinyl-CoA to 2-OG by KOR, it is possible that also POR enzyme is used in the reverse direction for anabolic purposes to support biosynthetic reactions. Additionally, the *F. acidiphilum* Y^T^ genome harbours two enzymes whose activity in the carboxylation direction might be involved in CO_2_ fixation: phosphoenol pyruvate carboxylase (PEPC) (FAD_1044) and NAD-binding malate oxidoreductase (malic enzyme FAD_0703) (Fig. 4).

Expression of genes for these four enzymes along with succinate and malate dehydrogenases was detected and quantified by real-time PCR. Prior to perform the RT-PCR assays we estimated the nucleic acids ratio in *F. acidiphilum* Y^T^ culture harvested after 4 days, which corresponded to the late exponential/early stationary growth phase. This value provides an indication of cellular RNA levels, i.e. metabolic state, and is independent of the number of cells examined. The estimated RNA ⁄ DNA ratio of 7.81 indicated that *F. acidiphilum* Y^T^ cells were actively metabolising at this state. Two housekeeping genes, *gyrB* and *rpl2* exhibiting constitutive levels of expression, were selected as standards to quantify the relative abundance of *F. acidiphilum* Y^T^ gene transcripts involved in both, TCA cycle and in anaplerotic CO_2_ assimilation (Table S1). Compared to *gyrB* transcripts, we detected a slightly higher transcription level of *rpl2* (the structural component of the large 50 S ribosomal subunit), which reflected the active metabolic state of *F. acidiphilum* Y^T^. Noteworthy, while comparable with expression levels of the references, relative amounts of *sdhA*, *sdhD* and *mdhI* transcripts were significantly reduced (40–200-fold) as compared to those of POR, KOR and malic enzyme. The PEPC transcripts were detected in quantities similar to those of *gyrB* (Fig. 4). As far as only PEPC catalyses irreversible carboxylation, the RT-PCR data confirm that direct carboxylation reactions do contribute to the inorganic carbon uptake by *F. acidiphilum* Y^T^ cells. We are aware that to confirm unambiguously the contribution of POR, KOR and malic enzyme to the total cellular carbon formation, more in-depth biochemical studies of anaplerosis are needed.

#### Transport mechanisms of *F. acidiphilum* Y^T^ are habitat-specific

To thrive in environmental settings with high concentrations of metals and metalloids (iron, copper, cadmium, zinc and arsenic) *F. acidiphilum* Y^T^ must possess the corresponding set of important transport mechanisms. Various genes coding for cation diffusion facilitator family, manganese/divalent cation and tellurium resistance ABC transporters were detected in the *F. acidiphilum* Y^T^ genome (Table S3). These transporters increase tolerance to divalent metal ions such as cadmium, cobalt, tellurium and zinc. Besides, they may provide essential cofactors like molybdate and tungsten for diverse enzymes.

*F. acidiphilum* Y^T^ is native to arsenic-rich environments, and to withstand the arsenite stress the genome encodes the ATP-dependent arsenite efflux pump. Genes for homologues of arsenite-sensitive regulator (FAD_1795) and arsenite efflux pump permease (FAD_1796) were found located in a single operon. A gene encoding for an arsenite efflux pump ATPase located distantly from the *ars* operon on the chromosome was also identified (FAD_1514). With regard to the phosphorus, the *F. acidiphilum* Y^T^ genome possesses one sodium-dependent phosphate transporter FAD_1510 and three inorganic phosphate:H^+^ symporters (FAD_1260, 1738, 1753). Previously we described the narrow specialisation of *F. acidiphilum* Y^T^ in uptake of organic substrates, highlighting that this strain was not capable of growth on any of tested compounds, including organic acids, alcohols and single amino acids, common sugars and related compounds^1^. The addition of yeast extract was observed to be essential for growth with the optimum concentration at 200 mg l^−1^. In concordance with these observations, *F. acidiphilum* Y^T^ genome is lacking genes for the transport and assimilation of common organic compounds other than amino acids, and has only one identifiable integral carbohydrate ABC transporter (FAD_1026–1028). Herewith, at least 7 oligopeptide/peptide ABC transporters and 17 transporters for amino acids were found. Additionally to this cluster of transporters, the *F. acidiphilum* Y^T^ genome harbours 48 genes for transporters belonging to the Major Facilitator Superfamily (MFS). Although poorly characterised, this large and diverse group of secondary transporters was found to participate in the export of structurally and functionally unrelated compounds and in the uptake of a variety of substrates including ions, amino acids and peptides^52, 53^. These MFS-affiliated genes were found to be located nearby genes for membrane and transposase IS4 family proteins, amino acids transporters or vitamins biosynthesis. Certain speculation on various possible functionalities might be done in this relation. *F. acidiphilum* Y^T^ MFS-related proteins exhibited the most significant similarity mostly to the counterparts from *Thermoplasmatales* known to possess highest number of MFS proteins among other *Euryarchaeota* (13 in average) according to http://supfam.org/SUPERFAMILY ^54^. In this context, the number of MFS-related genes found in *F. acidiphilum* Y^T^ genome (48) is within the range (in average, 40 per genome) for *Thermoplasmatales* that occupy the same or similar environments.

Consistently with the abundance of oligopeptide/peptide transporters, the genome of *F. acidiphilum* Y^T^ encodes 16 cytoplasmic and membrane-associated proteases and aminopeptidases, including tricorn protease FAD_0691 and its integrating factors F2 (FAD_0645) and F3 (FAD_0317) both possessing the aminopeptidase activity. In conjunction with these factors, tricorn protease can degrade oligopeptides in a sequential manner, yielding free amino acids^55^. Besides this sophisticated cell-associated proteolytic machinery, the genome of *F. acidiphilum* Y^T^ encodes three secreted acid proteases thermopsins (FAD_0679, 0833 and 1292). Thus, in concordance with physiology, the genome analysis indicates that *F. acidiphilum* Y^T^ has a metabolism specialised in efficiently converting proteins and peptides into amino acids. Noteworthy, the growth of the strain *F. acidiphilum* Y^T^ is strongly affected by the presence of yeast extract in amounts greater than 200 mg l^−1^ and is completely inhibited at concentrations greater than 2 g l^−1^. As we realised from the genome analysis, the membrane of *F. acidiphilum* Y^T^ is likely to be well supplied with numerous protein- and amino acid-transporting complexes determining exceptional nutrient-scavenging capabilities. If this is true, the sudden entry into the cytoplasm of an abundance of nutrients could overwhelm the respiratory metabolism with reducing power that would generate damaging level of toxic oxygen species, such as hydroxyl radicals and peroxides. Additionally, the *F. acidiphilum* Y^T^ cytoplasm would become overloaded by organic compounds, which could provoke the cell death by dehydration.

Additionally to the oligotrophic adaptation, the growth was not detected on the yeast extract alone without ferrous iron, which serves as the electron donor^1, 5^. Taken together, these data point to *F. acidiphilum* Y^T^ as an obligate peptidolytic chemomixotrophic oligotroph.

*F. acidiphilum* Y^T^ genome does not harbour any of known pathways of CO_2_ fixation, thus suggesting that the capability of *F. acidiphilum* to assimilate inorganic carbon^1, 51^ is probably a result of anaplerotic CO_2_ assimilation. An intriguing point to mention is the ubiquity of *F. acidiphilum* with their remarkable conservation of genomes. The ability to iron oxidation is solely characteristic to *Ferroplasmaceae* family members among all up to date cultivated and studied *Thermoplasmatales* archaea, which represents a certain advantageous/niche speciation trait and might contribute to the broad distribution of these archaea. This is in a strong contrast with *Picrophilus* or *Thermogymnomonas* spp. that have so far been detected exclusively on Japanese Isles.

One could speculate on another argument for the possible ancient origin of these archaea reflected in amino acid/peptides dependence, which was suggested to exist in first heterotrophs and which seems to be linked to sulfur-containing environments^56^. In concordance with this hypothesis, the genes for several protein families from an apparent ancient anaerobic core of the LUCA, e.g. for ferredoxin, several subunits of the Mrp-antiporter/hydrogenase family, numerous S-adenosyl methionine (SAM) dependent methyltransferases that rarely occur in aerobic prokaryotes^35, 36^, were found in the *F. acidiphilum* Y^T^ genome.

One of the interesting observations was a relatively high number of single nucleotide substitutions in the genome of *F. acidiphilum* Y^T^ after ~550 generations *in vitro*. We hypothesize that such a high mutation rate could be caused by faster growth rates under optimal conditions in the culture, which is untypical for these archaea in their real life in natural habitats where they tend to exhibit a remarkable genomic conservation even in geographically distant populations. Analysis of nucleotide substitutions suggests that the genome is prone to the further decrease in GC content. The ratios of synonymous to non-synonymous amino acid substitutions and the distribution of single nucleotide substitutions between coding and non-coding regions suggest that at least under optimal cultivation conditions, the neutral drift is a prevalent mode of the genome evolution *in vitro*. This hypothesis certainly requires a deeper experimental analysis with parallel cell lines run in continuous bioreactors and for a greater number of generations.

## Methods

### Reference strain and growth conditions

*F. acidiphilum* Y^T^ (DSM 12658^T^) was deposited to the DSMZ collection in 1998, and since then maintained in the laboratory, in 2008 the original isolate was retrieved from DSMZ for genome sequencing. *F. acidiphilum* Y^T^ was routinely grown on the Medium 9 K containing 25 g/l of FeSO_4_.7H_2_O, supplemented with 0.02% (w/vol) of yeast extract until the mid-exponential phase at as described previously^1^. For calculation of single substitution mutation rates, the 100-ml cultures were grown in Erlenmeyer flasks under optimal conditions^1^ since deposition of the strain to the DSMZ Strain Culture collection in 1998. As an inoculum, 10 ml of culture were used each time, with 164 repeated growth experiments. The final culture (2008) was subjected to the DNA extraction and sequencing. Isolation of DNA from both variants was conducted using Genomic DNA isolation kit (QIAGEN, Hilden, Germany).

### Sequencing and assembly

*De novo* sequencing data production of *F. acidiphilum* Y^T^ was conducted at the Liverpool University Genome Centre on a 454 FLX Ti (454 Life Sciences, Branford, CT, USA) using a standard library (34x) coverage. In addition, a library sequencing using Illumina 2000 was done at Fidelity Systems (short paired-end 400 bp, av. read 100, coverage x 639) and at the Sequencing Facility of the Helmholtz Centre for Infection Research (Braunschweig, Germany) (single end, 36 nt in average, x 233 coverage). Genome assembly and gap closure were performed by Fidelity Systems Ltd. (Gaithersburg, MD, USA) using Phred/Phrap and Consed^57–59^ have been operated for the final sequence assembly. DupFinisher^60^ was used for the correction of repeat mis-assemblies and 384 Sanger end-sequenced fosmids for the generation of a single scaffold (0.98 x coverage). For the full closure, a number of direct sequencing reactions has been conducted^61^. The genome was automatically annotated at Fidelity Systems (USA) using Fgenesb:2.0 and manually curated using GenDB v. 2.2.1 annotation system Ribosomal RNA genes were identified via BLAST searches^62^ against public nucleotide databases and transfer RNA genes using tRNAScan-SE v. 1.21^63^. The CRISPRFinder web service was used for the identification of CRISPRs^64^. The genome of *F. acidiphilum* Y^T^ variant grown in the lab for ~550 generations was sequenced using Illumina (average coverage: 233) and was further mapped on the assembled type strain genome. The genome sequence of *F. acidiphilum* Y^T^ has been deposited to the GenBank/EMBL/DDBJ with the accession number CP015363.

### RNA isolation and quantitative reverse transcription PCR analysis (Q-RT-PCR)

Q-RT-PCR was used to estimate the abundance of ten target genes transcripts (Table S1). *F. acidiphilum* Y^T^ cells were collected after 4 days (corresponding to onset of stationary phase) by centrifugation at 9000 rpm for 15 min of 15–25 ml culture and total RNA was immediately purified using miRVANA kit (Ambion). RNA samples were treated with Turbo DNA-free kit (Ambion Austin, TX, USA). To eliminate the residual DNA contamination present in the RNA preparations, a second DNase treatment (DNase I, Invitrogen) was included prior to complementary DNA (cDNA) production. cDNA synthesis was performed with SuperScript II Reverse Transcriptase (Invitrogen, Carlsbad, CA, USA) with 100 ng of total RNA and 2 pmol of Random Hexamer Primer (Thermo Fisher Scientific) according to the manufacturer’s instruction. All RT-PCR experiments were performed using an ABI 7500 Fast Real-Time PCR System thermocycler. Gene-specific primers and TaqMan® probes (Table S1) were designed using Primer Express^®^ software v.2.0 (Applied Biosystems, USA). 5′-6-FAM and 3′-BHQ1 labelled TaqMan® probes were obtained from Biomers (Germany). RNA samples were tested in triplicates along with “No Template Control” (NTC). The reaction mixtures for Taqman® Q-RT-PCR were as follows: 0.3 μM final concentration of each primer, 0.2 μM TaqMan probe, cDNA template equivalent to 1 ng of RNA starting material, 12.5 μl of 2X TaqMan® 5 Universal PCR Master Mix (PE Applied Biosystems) and ultrapure water added to the final volume of 25 μl. The reactions were performed under the following conditions: 2 min at 50 °C followed by 10 min at 95 °C, followed by 40 cycles of 15 s at 95 °C and 1 min at 60 °C. PCR specificity and product detection was checked by examining the temperature-dependent melting curves of the PCR products and by sequencing of cloned amplicons.

Generation of quantitative data by RT-PCR is based on the number of cycles needed for amplification-generated fluorescence to reach a specific threshold of detection (the Ct value). RT-PCR amplification was analysed using an automatic setting for the baseline and threshold values and using the relative standard curve method. Standards for all amplifications were prepared using known amounts of cloned target templates. Amplicons were generated by PCR amplification of the target genes from genomic DNA. The resulting amplicons were then purified using the Wizard SV Gel and PCR Clean–up System kit (Promega, Madison, WI, USA), and cloned in pGEM®-T Easy Vector System I (Promega). After cloning, plasmids were extracted using the QIAprep Spin Miniprep kit (Qiagen, Hilden, Germany) and DNA concentrations were measured using a Nanodrop® ND-1000 spectrophotometer. Standard curves were based on serial dilution ranging between 10^7^ and 10^1^ gene copies. Ct values were then automatically generated by software and exported for calculation of average Ct and standard deviation (SD) values of triplicates. The comparative method using *gyrB* mRNA as the normalizer was performed as described elsewhere^65^. For normalization based on multiple, most stably expressed housekeeping genes, we used a ribosomal *pL2* gene, which has equal to *gyrB* reaction efficiency (*E*) value of 1.90^66, 67^.

## Electronic supplementary material

Supplementary Info

Supplementary Table S2
