# Supplementary material for: Metabolic and evolutionary patterns in the extremely acidophilic archaeon Ferroplasma acidiphilum YT
Source: Sci Rep. 2017 Jun 16;7:3682. doi: 10.1038/s41598-017-03904-5 (PMC5473848; doi:10.1038/s41598-017-03904-5)
Supplement: Supplementary file 1 — Supplementary Info [file 41598_2017_3904_MOESM1_ESM.pdf]

Supplementary Information to the Manuscript

Metabolic and evolutionary patterns in the extremely acidophilic archaeon *Ferroplasma acidiphilum* Y^T

Olga V. Golyshina, Hai Tran, Oleg N. Reva, Sofia Lemak, Alexander F. Yakunin, Alexander Goesmann, Taras Y. Nechitaylo, Violetta LaCono, Francesco Smedile, Alexei Slesarev, David Rojo, Coral Barbas, Manuel Ferrer, Michail M. Yakimov and Peter N. Golyshin

Content:

Supplementary Table S1. *F. acidiphilum* Y^T genes examined by real-time reverse-transcription PCR in this study ………………………………………………………… Pg. 2

Supplementary Table S2. 116 single-nucleotide substitutions (separate Excel file)… Pg. 2

Supplementary Table S3. Transporters in *F. acidiphilum* Y^T ……………………………Pg. 3

Supplementary Figure S1. Average Nucleotide Identity (ANI) analysis……………… Pg. 6

Supplementary Figure S2. Overview of amino acid biosynthesis pathways………….. Pg. 7

Supplementary Table S1. *F. acidiphilum* Y^T genes examined by real-time reverse-transcription PCR in this study.

Locus tag	Gene	Name	Forward primer / Reverse primer	TaqMan®
**FAD_0161**	***rpl2***	**50S ribosomal protein L2**	F: 5’-ATACAGGAGCCCGAGTCACA-3’ R: 5’-TGTTGTGGTAGTGCCGTTGT-3’	5’-CACCGGGTAGAAATGCACCGTT-3’
**FAD_0374**	***gyrB***	**DNA gyrase B**	F: 5’-GCAGAGCTTCAAGGGGAGTT-3’ R: 5’-CGCCAACAGGTAATGCAGTA-3’	5’-CTAAACGGTGCTGAAGTAATCA-3’
FAD_0567	*porA*	2-oxoacid--ferredoxin oxidoreductase, α subunit	F: 5’-CAACCGGGCTTCCAACAAAGA-3’ R: 5’-GAGCACTATTCTTGGCATGT-3’	5’-AGATCTCAACCAGGTGCTTGGT-3’
FAD_0703	*morME*	malate oxidoreductase (malic enzyme)	F: 5’- ATGGCCAGGTGATGCAAAGA-3’ R: 5’- TGCATAGGAAGCAGCAACCA-3’	5’- CCAATCAGATAAATAATAGCAT-3’
FAD_0712	*korA*	2-oxoacid--ferredoxin oxidoreductase, α subunit	F: 5’- AGCAGGGCGTATCAGCTAAC-3’ R: 5’- AGGGTCATATGCCTGCCATT-3’	5’- AATGTTCGAGCCTTTCCCTGCA-3’
FAD_0713	*korB*	2-oxoacid--ferredoxin oxidoreductase, β subunit	F: 5’- AGATTGGTGCCCTGGATGTG-3’ R: 5’- AGCTGACAAAGCCTGTGTTA-3’	5’- TGACTTTGGTATAGTAAGTGCG-3’
FAD_0714	*sdhD*	Succinate dehydrogenase subunit D	F: 5’- AGCCCCATTTGCCACTGAAA-3’ R: 5’-TCAACCATAGTGCCGGAGAA-3’	5’- AATTAAACAATGCTATGTACCT-3’
FAD_0717	*sdhA*	Succinate dehydrogenase subunit A	F: 5’- TTCAACGCGCTGGAAACAAG-3’ R: 5’- TGAAGTGTGCACCCCTTGTT-3’	5’- ATATGCTATGGCTACTGGAGCA-3’
FAD_0718	*mdh*	Malate dehydrogenase	F: 5’- TTTGCACCCAGGACCCTAA-3’ R: 5’- CTGCAATGCGTATGCCATTA-3’	5’- AACAGATTAAGAAATATTCACC-3’
FAD_1044	*pepc*	phosphoenol pyruvate carboxylase	F: 5’- AGAAGGGAGCGGAAATTGCA-3’ R: 5’- GCACGTGGCAAACTAGCTTT-3’	5’- CTATTTGGATACTCCCGTAGTA-3’

Supplementary Table S2. 116 single-nucleotide substitutions (separate Excel file).

Supplementary Table S3. Transporters in *F. acidiphilum* Y^T

Sugar & polysaccharide transporters	
FAD_1026	ABC-type sugar transporter, ATPase component
FAD_1027	ABC-type sugar transporter, permease component
FAD_1028	ABC-type sugar transporter, permease component
FAD_1029	ABC-type sugar transporter, extracellular component
FAD_1459	sugar ABC transporter 1, permease protein
FAD_1460	sugar ABC transporter 1, ATP binding protein
AA transporters	
FAD_0093	ABC-type peptide transporter, permease component
FAD_0094	oligopeptide ABC transporter Dpp2, permease protein
FAD_0126	6TMS neutral amino acid family transporter
FAD_0228	amino acid transporter
FAD_0377	amino acid transporter related protein
FAD_0539	cationic amino acid transporter
FAD_0636	amino acid transporter
FAD_0655	ABC transporter peptide-binding protein
FAD_0659	ABC transporter permease. dipeptide/oligopeptide/nickel transport system
FAD_0802	amino acid/polyamine/organocation superfamily transporter
FAD_0803	aspartate/glutamate family transporter
FAD_0804	aspartate/glutamate family transporter
FAD_0805	amino acid/polyamine/organocation superfamily transporter

FAD_0853	ABC transporter peptide-binding protein
FAD_0914	amino acid/polyamine/organocation superfamily transporter
FAD_0915	amino acid/polyamine/organocation superfamily transporter
FAD_0919	ABC transporter substrate-binding protein (C-terminal fragment) peptide
FAD_1013	amino acid/polyamine/organocation superfamily transporter
FAD_1014	ABC-type peptide/opine/nickel family transporter ATPase
FAD_1015	ABC-type peptide/opine/nickel family transporter ATPase
FAD_1016	ABC-type peptide/opine/nickel family transporter
FAD_1017	ABC-type peptide/opine/nickel family transporter
FAD_1018	ABC-type peptide/opine/nickel family transporter
FAD_1069	amino acid transporter
FAD_1371	6TMS neutral amino acid family transporter
FAD_1571	amino acid transporter
FAD_1737	transporter probably aa
Major facilitator superfamily MFS multidrug efflux pumps and major facilitator superfamily permeases	
FAD_0778 _0801 _0803 _0815 _0839 _1106 FAD_1033 _1118 _1278 _1296 _1750_0006 _0143 _0213 _0472 _0497 _0561 _0684 _0768 _0796 _0822 _0844_0849 _0979 _0994 _0996 _1000 _1011 _1018 _1038 _1040_1047 _1048 _1129_1272 _1349 _1380 _1407 _1408 _1445 _1484 _1504 _1544 _1555 _1591 _1599 _1720 _1788 _1791	
Other transporters	
FAD_0049	stomatin family transporter
FAD_0249	cation diffusion facilitator family transporter, that increase tolerance to divalent metal ions such as cadmium, zinc, and cobalt

FAD_0250	cation diffusion facilitator family transporter, that increase tolerance to divalent metal ions such as cadmium, zinc, and cobalt
FAD_1045	ammonium transporter
FAD_1137	C4-dicarboxylate transporter
FAD_1177	ABC-2-type family permease
FAD_1178	ABC-2-type family permease
FAD_0071	VIT family Fe2+/Mn2+ transporter
FAD_1261	VIT family Fe2+/Mn2+ transporter
FAD_0016	transporter of sulfur-containing compounds
FAD_0264	transporter of sulfur-containing compounds
FAD_0848	transporter of sulfur-containing compounds
FAD_1341	transporter of sulfur-containing compounds
FAD_1460	nitrate/sulfonate/bicarbonate ABC transporter ATP-binding protein
FAD_1459	nitrate/sulfonate/bicarbonate ABC transporter ATP-binding protein
FAD_1458	nitrate/sulfonate/bicarbonate ABC transporter ATP-binding protein
FAD_1494	monovalent cation:proton antiporter-2 family transporter
FAD_1510	sodium-dependent phosphate transporter
FAD_1593	manganese/divalent cation transporter
FAD_1667	multidrug ABC transporter ATP-binding protein
FAD_1771	daunorubicin resistance ABC transporter
FAD_1800	Kef-type potassium transporter NAD-binding component

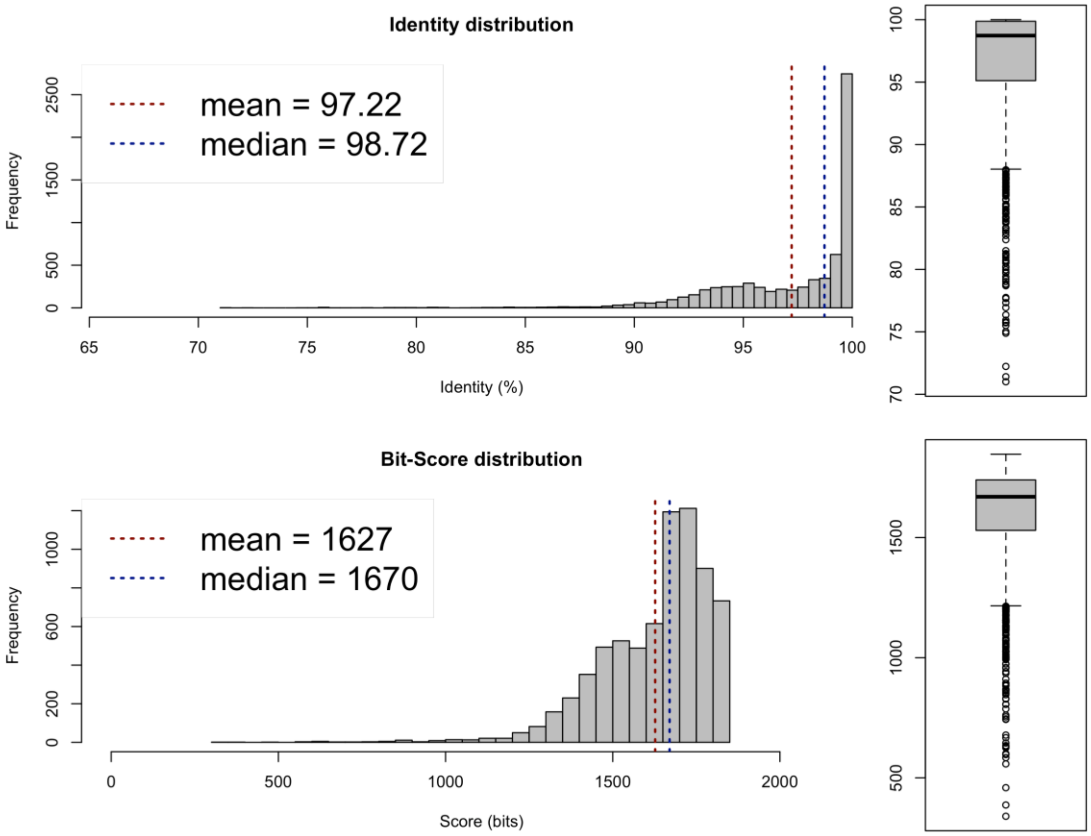

Supplementary Figure S1. Average Nucleotide Identity (ANI) analysis (http://enve-omics.ce.gatech.edu/ani/[1]) of genomes "*F. acidarmanus* fer1" and *F. acidiphilum* Y^T suggests ANI values above 95 %, which is the accepted cut off for separation of two species based on the whole-genome comparisons, suggesting that on the basis of their genomic data, *F. acidiphilum* Y^T and "*F. acidarmanus*" fer1 belong to the same species.

1. Goris, J., Konstantinidis, K. T., Klappenbach, J. A., Coenye, T., Vandamme, P. & Tiedje, J. M. DNA-DNA hybridization values and their relationship to whole-genome sequence similarities. *Int J Syst Evol Microbiol.* **57**, 81-91 (2007).

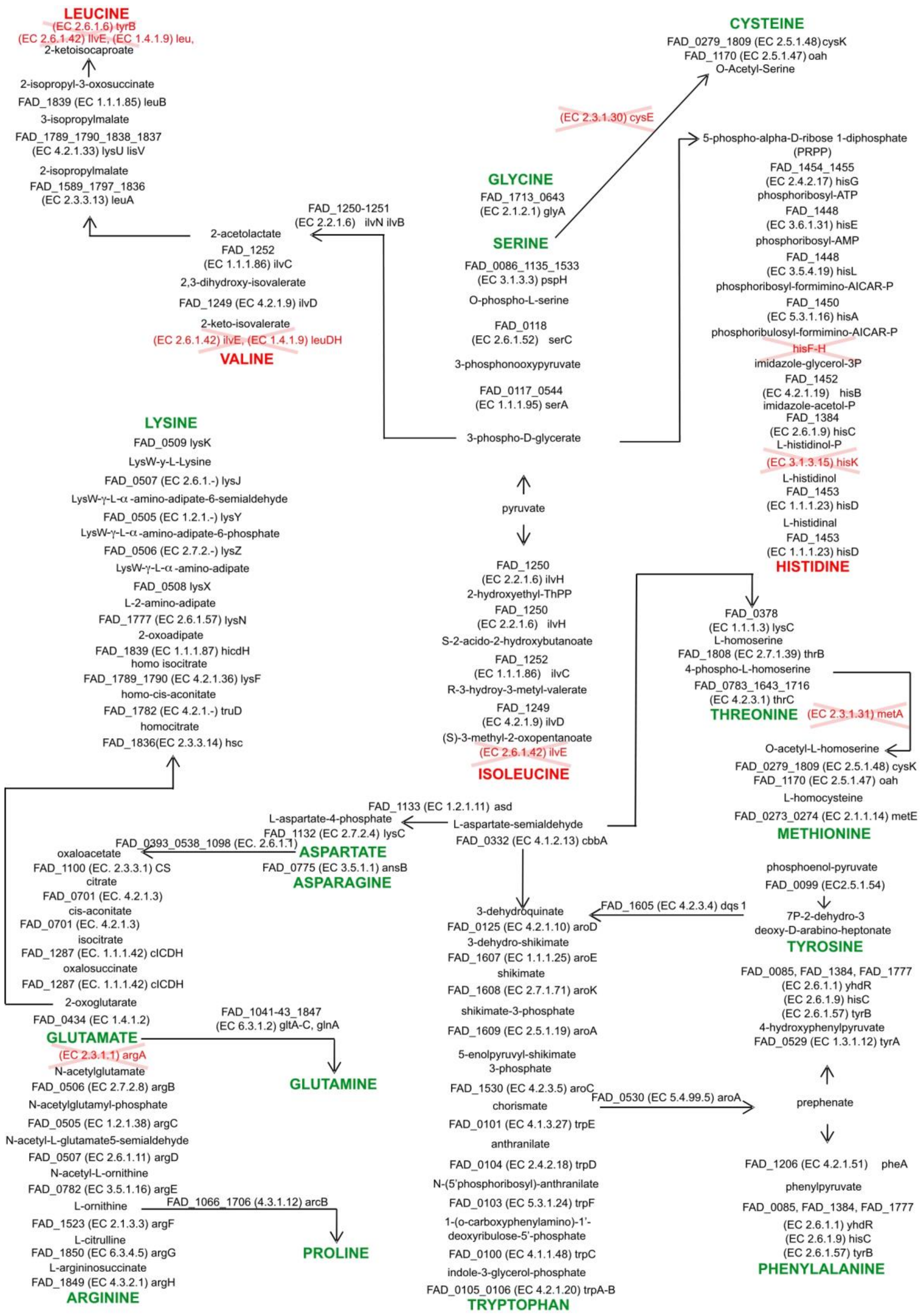

Supplementary Figure S2. Overview of amino acid biosynthesis pathways in the *F. acidiphilum* Y^{T} genome. Missing genes and reaction are indicated by red colour and crossed out. All amino acids with incomplete synthesis pathways are indicated in red. EC numbers are shown in parentheses.
